# Interpretation of TSH and T4 for diagnosing minor alterations in thyroid function: a comparative analysis of two separate longitudinal cohorts

**DOI:** 10.1186/s13044-022-00137-1

**Published:** 2022-10-10

**Authors:** Stig Andersen, Jesper Karmisholt, Niels Henrik Bruun, Johannes Riis, Paneeraq Noahsen, Louise Westergaard, Stine Linding Andersen

**Affiliations:** 1 Department of Clinical Medicine, Aalborg University, Aalborg, Denmark; 2 Department of Internal and Geriatric Medicine, Aalborg University Hospital, Aalborg, Denmark; 3 Department of Endocrinology, Aalborg University Hospital, Aalborg, Denmark; 4 Unit of Biostatistics, Aalborg University Hospital, Aalborg, Denmark; 5 Greenland Centre for Health Research, University of Greenland, Nuuk, Greenland; 6 Department of Clinical Biochemistry, Aalborg University Hospital, Aalborg, Denmark

**Keywords:** Thyroid dysfunction, Subclinical thyroid disease, Diagnosis, Thyroid function test interpretation, Biological variation

## Abstract

**Background:**

Minor alterations in thyroid function are frequent, and interpretation of thyroid function tests in the individual patient can be challenging. Furthermore, the choice of thyroid function test is debatable. To inform the debate, we performed a comparative evaluation of the variation in thyrotropin (TSH) and thyroxine (T4) in two different cohorts to illustrate the precision of TSH and T4 in the diagnosis and monitoring of thyroid dysfunction.

**Methods:**

A comparative analysis of two separate longitudinal studies previously surveyed with monthly blood sampling for one year among 35 subjects. Participants were included based on T4 within the reference range and TSH either within (euthyroid; *n* = 15) or above (subclinical hypothyroidism; *n* = 20) the laboratory reference range on two independent blood samplings before inclusion. Exclusion criteria were known thyroid disease or use of thyroid interfering medication. TSH and T4 in individual samples were measured in a single batch to prevent between-batch variation. The distributions TSH and T4 were compared among euthyroid and subclinical hypothyroid individuals, and bootstrap estimates were used to calculate area under the curve (AUC).

**Results:**

Collection of twelve, monthly blood samples in the 35 participants provided 420 samples, and data completeness was 100%. The mean TSH was 1.27/7.19 mIU/L and the mean total T4 was 106/85 nmol/L in euthyroid/subclinical hypothyroid participants. The subclinical hypothyroidism state deviated from the euthyroid by 20% for total T4 and by 466% for TSH. The overlap between the euthyroid and subclinical hypothyroid groups was 92.6% (389/420) for total T4 and 9.0% (38/420) of test results for TSH. The estimated AUC was 0.999 (95%-CI: 0.995; 1.00) for TSH and 0.853 (0.736; 0.935) for total T4. There was no confidence interval overlap between participant groups for TSH while there was a considerable overlap for total T4 (*p* < 0.001).

**Conclusion:**

The distributions of thyroid function tests illustrated how TSH outperforms T4 for detecting delicate differences in thyroid function in an individual. Thus, TSH was markedly better than T4 to discriminate between the subtle differences in thyroid function corroborating that TSH is the more sensitive and accurate index of thyroid function status in the individual patient.

## Background

Detection and monitoring of thyroid disorders require accurate measurement and interpretation of thyroid function tests [1–3]. While overt thyroid disease is easily recognised, interpretation of thyroid function tests can be challenging with minor alterations in thyroid function and with physiological strain [4–7]. Knowledge of the pattern of variation in thyroid function tests can be a clue to resolving apparently confusing test results [8, 9]. Thyroid function tests differ with repeated testing due to biological variation that adds to analytical and preanalytical variation [8, 9]. Biological variation shows distinct patterns, and knowledge of these inherent changes in thyroid function tests facilitates correct interpretation of test results [10, 11]. Additionally, knowledge of biological variation provides a statistical approach to the selection of the most appropriate test to aid the diagnostic process [12].

A recent meta-analysis of population studies found that thyroxine (T4) was more often correlated with all-cause mortality and morbidity than thyrotropin (TSH) [13]. These associations found in epidemiological studies tricked the authors to conclude that T4 is a better choice for diagnosing thyroid dysfunction in the individual patient [13]. This interpretation was based on an epidemiological approach and challenges recommendations to use TSH [1, 2] that follow the endorsed statistical approach [9, 12, 14].

This controversy inspired follow-up and led us to revisit data on variation in thyroid function tests [10, 11], which provided a clue to the interpretation of thyroid function tests in the individual [5–8, 14, 15]. Hence, we performed a comparative evaluation of the variation in TSH and T4 in two different cohorts to illustrate the precision of TSH and T4 in the diagnosis and monitoring of thyroid dysfunction to inform the debate on whether TSH or T4 is the better choice when managing the individual patient. Finally, the diagnostic power of TSH and T4 for detecting subclinical hypothyroidism was considered.

## Methods

Two separate groups of participants were included in the analysis, and the data had been collected separately. Both groups were surveyed to describe biological variation in thyroid function tests strictly following the recommended protocol [12]. The first group comprised 15 euthyroid individuals while the second group comprised 20 individuals with stable, untreated subclinical hypothyroidism (SH).

The study design and the sampling procedures were identical between the two groups, and they have been described in detail previously [10, 11]. In brief, 16 men were recruited for the euthyroid group and had thyroid function tests within the reference range prior to inclusion. One participant was excluded from the present analysis of euthyroid subjects due to the development of subclinical hyperthyroidism [10].

Participants in the group with stable, untreated SH were included based on two measurements of total T4 within the laboratory reference range and thyrotropin (TSH) between 5 and 12 mIU/L on repeated testing three months apart. Screening of subjects was based on routine laboratory test, which included total T4 at that time, and the upper limit of TSH was chosen to account for variation in TSH when aiming to include subjects with TSH consistently around 10 mIU/L. One of the 21 initial participants developed overt hypothyroidism, started Levothyroxine treatment, and was excluded from the analysis [11]. Further exclusion criteria for both groups were previous thyroid disease, thyroid affecting medication or diseases, change in any medication during the past three months, and pregnancy within the past year. No restrictions were made to the daily doings of the participants during the year of blood samplings. All participants were Caucasians and lived in Jutland, Denmark, an area with mild to moderate [16] and borderline [17] iodine deficiency during data collections.

All participants donated a monthly blood sample over one year, leading to 420 samples in the 15 euthyroid and 20 SH subjects as every participant completed the samplings. Samples were collected between 9 and 12 am, and plasma was separated and stored at -20 °C until analysis. When all the specimens were available for each of the two groups, analyses were performed with specimens from one individual analysed in random order using a single batch to eliminate between-run analytical variation. Data collection in the euthyroid group was terminated before data collection in the SH group, and the biochemical analysis was performed separately for the two groups.

In the euthyroid group, TSH was measured using immunochemiluminometric technique and a third-generation assay (LUMI-test, Brahms, Berlin, Germany). Total T4 was measured by a radioimmunoassay (Amerlex-M T4 RIA Kit, Johnson & Johnson, Cardiff, UK) and T3-uptake for calculation of free thyroxine index using reagents from Farmos Diagnostica (Oulunsalo, Finland) [10].

In the SH group, TSH and free T4 were measured using the immunochemiluminometric technique on Modular Analytics E170 (Roche Diagnostics, Mannheim, Germany) [11].

The performance of the assays has been described in detail previously [10, 11]. Reference ranges applied at our laboratory were 0.3–4.5 mIU/L for TSH, 60–140 nmol/L for total T4, 70–140 nmol/L for free T4 index [10], and 12–22 pmol/L for free T4 [11].

### Statistics

Frequencies, percentages, means, and standard deviations (SD) were used to describe the participants. The significance of differences was tested using non-paired t-test. Non-parametric ROC curves for TSH and total T4 were presented. Tie- and bias-corrected AUCs with bootstrapped 95% confidence intervals were compared. TSH and T4 were categorised according to the reference intervals. Sensitivity, specificity, positive- and negative predictive values were calculated for these categories. A *p*-value of less than 0.05 was considered significant. Stata Statistical Software, Release 17 (Stata Corp LLC, TX, College Station, 2021) was used for the analysis.

## Results

The 12 monthly sample collections were completed in all 35 participants with no missing data. The two groups differed by age, gender, and smoking habits. Participants differed by TSH and T4 with higher TSH and lower T4 in the SH group (Table 1). Thus, the true thyroid state was confirmed by the mean of the 12 repeated measurements of serum TSH and T4. A 19.6% lower total T4 was paralleled by a 466% higher TSH among SH patients compared with the euthyroid group. No TSH measurement was above the upper limit of the population-based reference range in the euthyroid group, and 100% of test results conformed to the euthyroid state. In the SH group, 87% of TSH test results were above the upper limit of the reference range. For total T4, 99% and 97% of test results were within the population-based reference range among euthyroid and SH subjects.

**Table 1:** Participants in the two groups differing by thyroid function. The euthyroid participants had TSH and T4 within the population-based reference range while the participants with subclinical (mild) hypothyroidism had elevated TSH and T4 within the reference range in two separate blood samples prior to inclusion. Twelve monthly blood samplings were done in all participants

	**Euthyroid**	**Mildly hypothyroid**^b^	***P*-value**
Number of participants	15	20	
Number of samples	180	240	
Age, range (mean;SD), years^a^	26–53 (39;10)	27–78 (57;12)	< 0.001
Sex, men, n	15	2	< 0.001
Weight, mean (SD), kg^a^	81 (11)	77 (14)	0.32
Current smoker, n	11	4	0.02
TSH, mean (SD), CV%	1.27 (0.56), 44.0	7.19 (3.03), 42.1	< 0.001
0.3–4.5 mIU/L, n (%)	180 (100)	31 (12.9)	
> 4.5 mIU/L, n (%)	0 (0.0)	209 (87.1)	
TT4, mean (SD), CV%	106.4 (20.8), 19.6	85.5 (16.2), 18.9	< 0.001
60–140 nmol/L, n (%)	179 (99.4)	233 (97.1)	NS
< 60 nmol/L, n (%)	1 (0.6)	7 (2.9)	
fT4 index, mean (SD), CV%	102.4 (20.9), 20.4		
70–140 nmol/L, n (%)	176 (97.8)		
< 70 nmol/L, n (%)	4 (2.2)		
fT4, mean (SD), CV%		13.0 (2.4), 18.6	
12 + pmol/L, n (%)		188 (78.3)	
< 12 pmol/l, n (%)		52 (21.7)	

*TSH*: Thyrotropin, *TT4*: Total thyroxine, *fT4*: Free thyroxine

^a^ *SD* Standard deviation

^b^ One participant had subsequent measures of TSH and T4 within the reference range. Without this participant mean TSH was 7.39 mIU/L while mean T4 was unaltered

One participant classified with SH according to the inclusion criteria had all TSH and total T4 test results within the reference ranges during the study year. The test results reported adhere to inclusion criteria with this participant included in the SH group. Omitting this participant caused the mean TSH to be 7.39 mIU/L and an increase in the number of TSH measurements above the upper limit of the reference range in the SH group from 87 to 92%. The mean total T4 was unaltered at 85.5 nmol/L, as was the fraction of T4 test results within the reference range.

Table 2 lists sensitivity, specificity, positive- and negative predictive values for TSH, total T4, and free T4 calculated using the data provided in Table 1. The positive predictive value of TSH was 100% compared to 87.5% for total T4, and the negative predictive values were 85.3% for TSH and 43.3% for total T4. The free T4 estimates showed higher sensitivity and higher positive predictive value compared to total T4.

**Table 2:** The ability of thyroid function test to detect deviations from the euthyroid state among euthyroid participants and patients with subclinical (mild) hypothyroidism. Calculations are based on the measurements listed in Table 1

	**TSH**	**TT4**	**fT4 index**	**fT4**
**Laboratory reference range:**	**0.3–4.5 mIU/L**	**60–140 nM**	**70–140 nM**	**12–22 pM**
Samples *within* the reference range:				
Euthyroid, % (n)	100 (180)	99.4 (179)	97.8 (176)	
Mildly hypothyroid, % (n)^a^	12.9 (31)	97.1 (233)		78.3 (188)
Sensitivity, %	87.1	2.9	21.7	
Specificity, %	100	99.4	98.9	
PPV, positive predictive value, %	100	87.5	96.3	
NPV, negative predictive value, %	85.3	43.4	48.8	

*TSH* Thyrotropin, *TT4* Total thyroxine, *fT4* Free thyroxine

^a^ One participant had subsequent measures of TSH and T4 within the reference range. Without this participant, 8.3% of TSH and 96.9% of T4 measurements were within the population-based reference range in this group

**Fig. 1:**
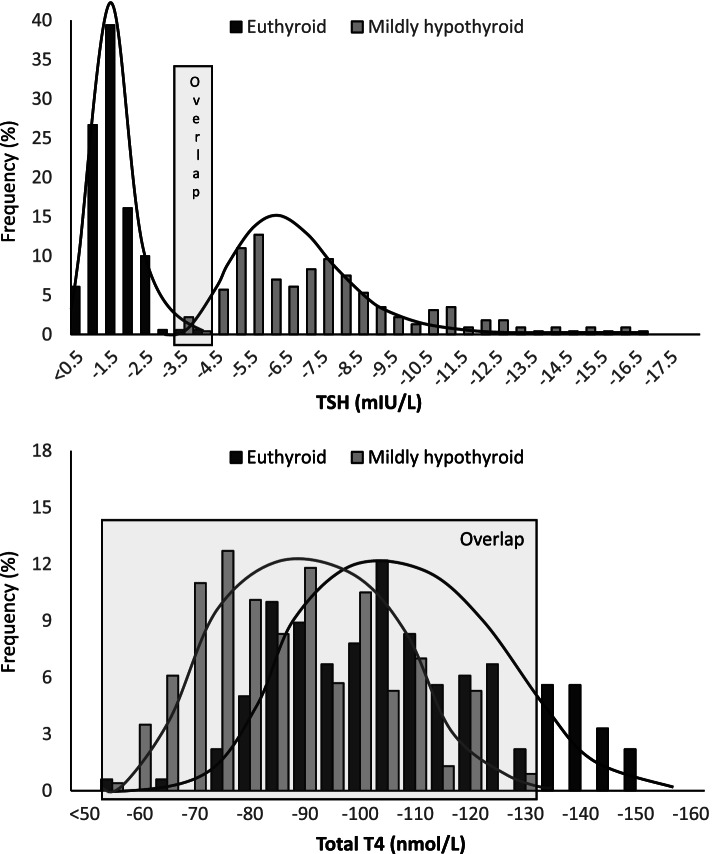
The distribution of twelve, monthly measurements of TSH and T4 among 35 subjects who were either euthyroid (black bars) or had mild thyroid hormone deficiency (shaded bars). The obvious overlap between the two groups for T4 (lower bar) receded for TSH (upper bar). This is in keeping with an amplified response in TSH to minor differences in T4

Figure 1 illustrates the distribution of the repeated test results. Differences in mean and in distribution between the two participant groups were markedly more pronounced for TSH than for total T4. The overlap between test results among the euthyroid and SH groups was limited for TSH (upper bar, narrow box) while it was broader for total T4 (lower bar, wide box): the overlap was 9.0% (38/420) of test results for TSH and 92.6% (389/420) for total T4. Including only the 19 participants with confirmed SH, the overlap was narrower for TSH, 2% (8/408), while it remained at 92.4% (377/408) for total T4.

Bootstrap estimates based on 1000 replications showed an estimated AUC of 0.999 (95%-CI: 0.995; 1.00) for TSH and 0.853 (0.736; 0.935) for total T4 conforming to a markedly better performance of TSH compared to total T4 for defining the true thyroid function state of an individual. There was no confidence interval overlap between participant groups for TSH, while there was a considerable overlap for total T4 (*p* < 0.001).

## Discussion

Our data from monthly blood sampling for one year in two separate groups either diagnosed with stable, untreated subclinical hypothyroidism or being euthyroid illustrate a substantial overlap between groups for T4, but not for TSH. This finding corroborates that measurement of TSH is markedly more sensitive and accurate than T4 for detecting minor alterations in thyroid function in individuals with intact hypothalamic-pituitary function.

The two groups included in our analysis differed by an average of 20 nmol/L for total T4 and 5.92 mIU/L for TSH. This 20% lower total T4 was accompanied by a 466% higher TSH corresponding to an exponential amplification of response in TSH to linear changes in T4. The magnitude of amplification is similar to that shown by Spencer and colleagues in a cross-sectional design [18], and in keeping with the marked pituitary response to fine adjustments in thyroxine replacement therapy reported in treated hypothyroid patients by Carr et al. [19]. Thus, our results conform to, and add an illustration of, the consequences of the log-linear association between TSH and T4 using data on biological variation in euthyroid and SH subjects. Our present report is a new approach to illustrate this phenomenon, and the findings support clarity in interpretation of thyroid function tests when results are challenging.

The fact that clinical consequences can be identified even with thyroid function tests within the reference range [20] emphasises their limitations [5, 6, 9, 15], and supports that the better correlation between T4 and all-cause mortality and morbidity in populations has limited relevance for the management of thyroid dysfunction in the individual patient [21]. The lack of sensitivity of population-based reference ranges to detect deviations in thyroid function tests from the narrow individual setpoint has been established in several settings [10, 11, 22–28]. It highlights the importance of the amplified response in TSH to minor changes in T4 to detect true changes in thyroid function. Using the average participant as an example, a 20% lower total T4, e.g., a decrease from 100 to 80 nmol/L, will go undetected within the population-based reference range, while the parallel amplified 400% rise in TSH, e.g., from 2.5 to 10 mIU/L, will ensure that TSH departs from the population-based reference range. Hence, mild thyroid dysfunction becomes obvious from TSH in the patient with intact hypothalamic-pituitary function.

The interplay of the stimulatory effect of TSH and inhibitory effect of thyroid hormones on the pituitary and the hypothalamus regulates both amplitude and frequency of secretory bursts from the normal pituitary gland [9, 29, 30] to maintain thyroid hormones within the narrow limits in the individual [8–10, 31]. The amplified response in TSH to small changes in T4 is a clue to the power of this mechanism. The efficacy of the feedback system with tight hormonal control was illustrated in a recent trial of the intake of sushi- and seaweed meals followed by daily thyroid function tests. The excessive iodine intake blocks the secretion of thyroid hormones from the thyroid gland, and the response was a 50% increase in TSH by day 2, coming down to pre-meal values on day 3, while the parallel T4 secretion was unaltered [32]. Similar response in TSH has been found in different populations [33], and the ability of TSH to maintain thyroid hormone levels within a narrow range demonstrates the power and responsiveness of the hypothalamic-pituitary-thyroid axis. Furthermore, it makes sense to have a robust mechanism with the capacity to secure the thyroid hormone levels required during physiological strain [7, 26, 28, 34]. Accordingly, the TSH response is powerful and sensitive for detecting small changes in T4, making it a reliable marker of mild thyroid dysfunction.

In our data, the exponentially amplified TSH-response to differences in T4 caused 87% of TSH measurements to be outside the reference range in SH participants, while this was the case for 3% for total T4. Thus, the sensitivity for detecting thyroid dysfunction was around 87% for TSH and 3% for total T4. The difference in overlap between groups was less than 10% for TSH and more than 90% for T4. This difference illustrates the higher sensitivity of TSH compared to T4 to detect mild thyroid dysfunction.

While it still needs to be settled whether to treat or not to treat subclinical thyroid dysfunction, there is consensus on the need to monitor thyroid function in these patients by regular measurements of thyroid function tests [1–3, 34]. Monitoring of such subtle deviations in thyroid function tests is highly dependent on accurate measurement and interpretation of thyroid function tests as clinical manifestations are often none-specific if present, particularly in old age [35], but still accompanied by the raised risk of diseases attributable to thyroid dysfunction [20]. The likelihood of detecting minor deviations in thyroid function in the individual is thus higher when evaluation is based on TSH rather than T4.

The finding in the recent review and meta-analysis that T4 levels were stronger associated with disease outcomes than TSH led the authors to recommend reconsidering the TSH-based diagnostic approach to thyroid function [13]. Their analysis was based on associations in populations, and care should be taken when using epidemiological data on an individual patient level. Hence, the recommended methodology for selecting the best specimen for defining a disease state in the individual patient is based on a statistical approach [12, 21]. In the present report, we addressed this controversy and added an illustration by reassessing our data to inform if TSH or T4 is the best test for diagnostic and monitoring purposes. Our findings corroborate the reports complying with standards on the generation of numerical data recommended for selecting the best specimen that TSH is the most sensitive indicator of thyroid function available.

It is a limitation that the analysis of TSH, total and free T4 were performed separately for the SH and euthyroid participants. Also, the estimates of free T4 were performed by two different methods. Due to the use of different assays, we performed comparisons for only TSH and total T4 with similar population-based reference ranges, and we restricted the use of estimates of free T4 to calculate the fraction of free T4 outside the assay-specific reference ranges. Another limitation was the difference in age and gender between the two groups. Participants had slight iodine deficiency, which may cause TSH levels to decrease with age and hence diminish the differences between participant groups. We saw no differences with age or gender. In addition, variance is essential to the performance of thyroid function tests as evaluated, and there is no evidence to suggest an influence on variation within the age span of the study or with gender. Still, results should be interpreted with caution outside the age span of participants as pituitary responsiveness decreases with high age [36]. Our analysis rested on the assumption of random, independent fluctuations around a homeostatic set point, which has been reported previously [30], and the time interval between specimen collections of four weeks was sufficient to allow an effect of disturbances to the homeostatic system to decay. A strength of our study was the collection of data in strict accordance with the protocol for the generation of numerical data and application of data on biological variation. The number of specimens collected, the number of subjects studied, and the time interval between specimen collections exceeded minimum recommendations [12], and the reliability of our results was supported by complete data collections. We included otherwise healthy individuals maintaining their usual lifestyles for the duration of the study. Finally, the assay protocol prevented results from being confounded by between-batch analytical variation.

## Conclusion

A 20% difference in total T4 was accompanied by a 466% difference in TSH. This amplified response in TSH to small changes in T4 led to separate distributions in TSH while total T4 showed considerable overlap between the two participant groups. Hence, the sensitivity and specificity of TSH vastly exceed that of T4 for detecting devious deviations in thyroid function. Our data illustrate the higher diagnostic power of TSH compared to T4 in the individual patient, emphasise the strength of the statistical above the epidemiological approach to biochemical parameters for individual patient diagnosis, and ease the interpretation of thyroid function tests with minor alterations in thyroid function. Thus, TSH is illustrated to be the most sensitive and accurate index of thyroid status of an individual when the hypothalamic–pituitary function is intact.

## Data Availability

The data generated and analysed during the current study are not publicly available due to personal identification issues but can be made available from the corresponding author on reasonable request.
